# Probiotics and amelioration of periodontitis: significant roles of *Lacticaseibacillus casei* DS31

**DOI:** 10.3389/fmicb.2025.1715664

**Published:** 2025-12-12

**Authors:** Yang Liu, Peng Wu, Yizhen Li, Xiaofeng Chen, Ling Wang, Yanjun Su, Lianzhong Luo, Yihuang Cai, Qinmiao Huang, Xu Tang

**Affiliations:** 1 Third Institute of Oceanography, Ministry of Natural Resources, Xiamen, China; 2 Fujian Key Laboratory of Island Monitoring and Ecological Development (Island Research Center, MNR), Pingtan, China; 3 Stomatological Hospital of Xiamen Medical College, Xiamen, China; 4 School of Pharmacy and Pharmaceutical Sciences, Xiamen Medical College, Xiamen, China; 5 School of Stomatology, Xiamen Medical College, Xiamen, China; 6 Fujian Huijing Biological Technology Co., Ltd., Zhangzhou, China

**Keywords:** Lacticaseibacillus casei, periodontitis, Porphyromonas gingivalis, biofilm, rats

## Abstract

**Background:**

Periodontitis, a chronic gum disease caused by *Porphyromonas gingivalis* infection, if left untreated, can result in tooth loss, alveolar bone loss, halitosis and other oral health complications.

**Methods:**

To investigate the preventive and therapeutic effects of *Lacticaseibacillus casei* DS31 from the large yellow croaker (*Larimichthys crocea*) on periodontitis, three experimental models—biofilm, cellular, and animal models—were established to systematically evaluate its efficacy. First, we sought to clarify the effect of DS31 against *P. gingivalis* biofilm. Then, the investigation entailed a comprehensive examination of the immunomodulatory effects of heat-inactivated probiotics on inflammation-inducing cells. Finally, the impact of probiotics on gingival tissue and alveolar bone was evaluated using an established periodontitis rat model.

**Results:**

The results demonstrated that bacteria suspension or cell-free supernatant of *L. casei* DS31 effectively inhibited *P. gingivalis* biofilm formation and eradicated existing biofilms, thereby reducing the secretion of pro-inflammatory cytokines (TNF-α, IL-6 and IL-1β) and inflammatory mediators (NO). Microcomputed tomography (micro-CT) and histopathological analysis revealed that supplementation with BS-DS31 or CFS-DS31 mitigated alveolar bone loss and increased bone mineral density in the experimental animals. The secretion of inflammatory factors (TNF-α, IL-1β, IL-6 and IL-8) in the gingival tissue of the rats was reduced.

**Conclusion:**

*Lacticaseibacillus casei* DS31 demonstrates significant potential for alleviating periodontitis and could serve as a promising probiotic candidate for incorporation into functional foods and oral health therapeutic applications.

## 1 Introduction

Periodontitis is a chronic, multifactorial inflammatory disease associated with dysbiotic dental plaque biofilms and characterized by progressive destruction of the tooth-supporting apparatus. Its primary clinical features include loss of periodontal tissue support, as evidenced by clinical attachment loss (CAL) and radiographically confirmed alveolar bone loss, as well as the presence of periodontal pocketing and gingival bleeding (Papapanou et al., 2018). Extensive research has established a robust association between periodontitis and various systemic conditions, including diabetes mellitus, cardiovascular diseases, dyslipidemia, stroke and osteoporosis (Wang and McCauley, 2016; Minty et al., 2019; Bassani et al., 2023; Zheng et al., 2023). Periodontitis imposes a significant burden on global public health (Qiao et al., 2018; Yang et al., 2023).

The oral microbiota consists of more than 700 distinct microbial species, which form complex biofilms that are essential for health. Disruption of microbial homeostasis in periodontal tissues can lead to the overgrowth of pathogenic organisms within these biofilms, resulting in periodontitis and eliciting host immune responses (Cui et al., 2025). Subgingival microbial dysbiosis—characterized by an increased abundance of Periodontal Red Complex Pathogens, such as *Porphyromonas gingivalis*, *Treponema denticola*, and *Tannerella forsythia*—plays a pivotal role in the initiation and progression of periodontal disease (Yadalam et al., 2023). Furthermore, oral dysbiosis may exert systemic effects via immune-mediated mechanisms. Regional immunity in periodontal tissues, mediated by neutrophils, T helper 17 cells, and various immune-related cytokines, is critical for preserving periodontal homeostasis and responding to microbial disturbances. Therefore, the microbiome is widely recognized for its critical role in maintaining overall bodily health (Meng et al., 2020; Réthi-Nagy and Juhász, 2024). Specifically, the oral microbiome significantly contributes to the oral barrier. An imbalance within this microbial community, characterized by an increased proportion of anaerobic Gram-negative bacteria, can lead to periodontitis (Ai et al., 2022; Chew et al., 2025). *P. gingivalis*, a key pathogenic bacterium in periodontitis, forms a dense biofilm on tooth surfaces (Guo et al., 2020). Its virulence factors, including lipopolysaccharide, proteases, and fimbriae, not only enhance bacterial colonization and facilitate the expansion of the surrounding microbial community but also promote coaggregation with other bacteria and the formation of dental biofilm (Xu et al., 2020). Moreover, these virulence factors modulate various host immune components, subverting the immune response to either facilitate bacterial evasion from clearance or induce an inflammatory environment (Abdi et al., 2017). In biofilm form, bacterial resistance to antibacterial substances is 10 to 1000 times greater than that of planktonic bacteria. And the gene expression profiles differ significantly from those of planktonic bacteria (Romero-Lastra et al., 2017).

Current treatments for periodontitis predominantly involve scaling and root planning therapy as well as antibiotic administration (Valenza et al., 2009; Ardila and Bedoya-García, 2022; Wang et al., 2024). However, these methods are insufficient in preventing disease recurrence and may contribute to the development of antibiotic resistance (MacLean and San Millan, 2019; Cobb and Sottosanti, 2021; Chen et al., 2025). Due to the limitations of existing therapies, there is an urgent need to explore safer and more patient-friendly alternatives that can potentially replace conventional treatments. Therefore, the development of innovative preventive and therapeutic strategies is of critical importance.

Probiotics are defined as living microorganisms that can exert beneficial effects on the host when administered in adequate amounts (Cremon et al., 2018). Probiotics are predominantly consist of lactic acid bacteria (LAB), including *Lactobacillus* spp. (Mokoena, 2017). Probiotics are directly edible strains that can serve as a source of functional food, maintaining host health primarily by maintaining a balance between beneficial strains, secreting antibacterial compounds, and regulating immune responses (Abbasiliasi et al., 2012; Gryaznova et al., 2022; Sugajski et al., 2022). In addition, LAB have been actively studied for their effects, such as improving cardiac function, reducing cholesterol level and mitigating diabetes mellitus (Silva-Cutini et al., 2019; Wang G. et al., 2020; Wan et al., 2023). In recent years, researchers have also found that probiotics can also regulate the oral microbiome. Leading to increased interest, probiotic therapy is increasingly being used to prevent and treat dental caries, halitosis, periodontitis, and other chronic oral infectious diseases (Bustamante et al., 2020; Wang et al., 2022; Robo et al., 2024). Currently, probiotics with preventive and therapeutic potential against periodontitis are primarily derived from terrestrial sources, whereas probiotics from marine sources have not yet been reported.

The objective of this study is to investigate the preventive or therapeutic effects of *L. casei* DS31bacterial suspension or its supernatant on periodontitis. To investigate the prevention and treatment of periodontitis, we established biofilm models to evaluate the efficacy of probiotics. Subsequently, we developed a periodontitis cell model using macrophages cell line RAW264.7 to assess the anti-inflammatory efficacy of *L. casei* DS31. Ultimately, a periodontitis rat model was established to demonstrate the therapeutic potential of *L. casei in vivo*. This strain exhibits inhibitory effects against periodontitis-causing pathogens and demonstrates potential for the prevention and treatment of periodontitis. The summarized results provide suitable information in the field of the host-microbiota-pathogen interaction.

## 2 Materials and methods

### 2.1 Preparation of experiment

The *L. casei* DS31 strain was cultured in de Man, Rogosa, and Sharpe (MRS) broth (Huankai Microbial Sci. & Tech, Guangdong, China) for 16 h at 37 °C and adjusted to a density of 10^6^–10^8^ CFU/mL. The adjusted culture medium was inoculated with 1% in fresh MRS broth and further cultured for 48 h. The bacterial suspensions of *L. casei* DS31 (BS-DS31) was harvested. Subsequently, the bacterial suspensions were centrifuged at 9500 rpm for 30 min and filtered to obtain a cell-free supernatant (CFS-DS31).

*Porphyromonas gingivalis* (ATCC 33277) was obtained from the China General Microbiological Culture Collection Center and was propagated on Columbia blood agar (Hopebio Company, Qingdao, China) at 37 °C in an anaerobic incubator (85% N_2_, 10% H_2_, and 5% CO_2_) for 48 h. Next, Tryptic Soy Broth (TSB) broth (Hopebio Company) was inoculated with a *P. gingivalis* culture (1%, v/v) followed by incubation at 37 °C for 72 h.

### 2.2 Cell culture and differentiation

RAW264.7 cells (murine macrophages) were cultured in Dulbecco’s Modified Eagle Medium (DMEM; Gibco, Grand Island, NY, USA) supplemented with 10% heat-inactivated fetal bovine serum (FBS; Gibco) and 1% of penicillin-streptomycin solution (P/S, Gibco). Cultures were incubated at 37 °C in a humidified atmosphere containing 5% CO_2_.

### 2.3 The effect of DS31 against *P. gingivalis*

#### 2.3.1 Biofilm formation assay

*Porphyromonas gingivalis* cultures that incubated for 72 h at 37 °C were adjusted to 10^6^ CFU/mL through dilution in TSB broth. Next, the DS31 (BS-DS31 or CFS-DS31) and *P. gingivalis* were added to upper and lower chambers of 24-well transwell plates (0.4 μm, Corning, NY, USA), respectively, at a ratio of 1:1 (v/v) (Luan et al., 2022). After the incubation of transwell plates for 48 h at 37 °C, the fluid culture medium within the lower chamber was gently removed and discarded; then, the *P. gingivalis* biofilm at the bottom of each well was gently washed three times with PBS to remove any planktonic bacteria. Thereafter, *P. gingivalis* biofilm in each lower chamber was fixed using a 99% (v/v) methanol solution at 4 °C for 30 min and subsequently air-dried at room temperature. Next, Gram staining kit was added to each lower transwell chamber. Thereafter, *P. gingivalis* biofilm structure was observed under a microscope (XD-202, Jiangnan, Nanjing, China).

#### 2.3.2 Biofilm eradication assay

*Porphyromonas gingivalis* cultures that had been incubated for 72 h at 37 °C was adjusted to 10^6^ CFU/mL through dilution in TSB broth. Next, *P. gingivalis* was added to upper and lower chambers of 24-well transwell plates (Luan et al., 2022)(0.4 μm, Corning, NY, USA). After the incubation of transwell plates for 120 h at 37 °C, the fluid culture medium within the lower chamber was gently removed and discarded; then, the *P. gingivalis* biofilm at the bottom of each well was gently washed three times with PBS to remove any planktonic bacteria. The BS-DS31 or CFS-DS31 was added to the biofilm for 48 h. Subsequently, *P. gingivalis* biofilm in each lower chamber was fixed in 99% (v/v) methanol solution at 4 °C for 30 min and dried at room temperature. Next, Gram staining kit was added to each lower transwell chamber. Thereafter, *P. gingivalis* biofilm structure was observed under a microscope.

### 2.4 Nitric oxide (NO) production

The production of nitric oxide (NO) was quantified in *P. gingivalis* LPS-stimulated RAW 264.7 cells using assay kits to measure levels of each compound (Thermo Fisher Scientific Inc., Waltham, MA USA). RAW 264.7 cells were seeded in 96-well plates at a density of 2 × 10^4^ cells/well and incubated overnight at 37 °C The cells were treated with DMEM (FBS-free) containing 1 μg/mL of *P. gingivalis* LPS (PgLPS; InvivoGen, San Diego, CA, USA), and then treated with 1% heat-killed BS-DS31 or CFS-DS31, further incubated for 24 h. Thereafter, the culture medium of each group (90 μL) and fresh Griess reagent (10 μL) were mixed in a new plate and incubated at 37 °C for 30 min. The absorbance was measured at 548 nm using a microplate reader (Molecular Devices, Shanghai, China). NaNO_2_ was used as the standard to quantify NO.

### 2.5 Cytokine and inflammation assays

To assess *P. gingivalis* anti-inflammatory activity, RAW264.7 cells were treated with heat-killed BS-DS31 or CFS-DS31 for 1 h and then 1 μg/mL of *P. gingivalis* LPS (InvivoGen, San Diego, CA, USA) was added to cells followed by incubation of stimulated cells for 24 h (37°C, 5% CO_2_, 90% relative humidity) (Luan et al., 2022). The effects of *L. casei* DS31 on the production of TNF-α, interleukin-6 (IL-6), interleukin-1β (IL-1β) were assessed in *P. gingivalis* LPS-induced RAW 264.7 cells using enzyme linked immunosorbent assay (ELISA) kits (Shanghai Enzyme linked Biotechnology Co., Ltd., Shanghai, China).

### 2.6 Animal experiment design

**FIGURE 1:**
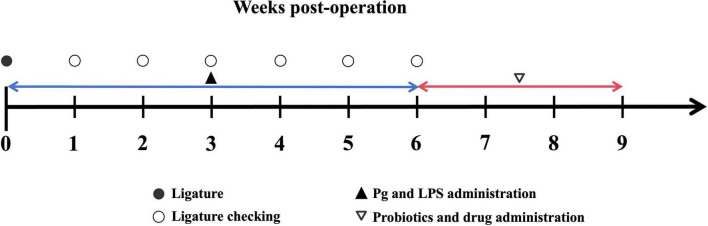
The experimental timeline of periodontitis model.

All protocols were approved by the Institutional Committee on the Care and Use of Animals of the Third Institute of Oceanography, Ministry of Natural Resources. A total of 15 8-week-old both sexes Wistar rats weighing 200–210 g (SLAC Laboratory, Shanghai, China). The rats were maintained under a 12-h light/dark cycle at a temperature of 22–24 °C. The experimental timeline is shown in Figure 1.

After a 1-week adaptation period, 15 rats were randomly divided into 2 groups: three rats in the normal control (NC) group and twelve rats in the Periodontitis disease (PD) groups. During the 6-week modeling period, the PD groups were randomly divided into four groups: Periodontitis disease (Model), DS31 bacterial suspension (BS-DS31), DS31 cell-free supernatant (CFS-DS31) and positive control (PC) groups. In the following 3 weeks, apart from the model group and the healthy group, three experimental groups were given BS-DS31, CFS-DS31 and compound tinidazole.

**FIGURE 2:**
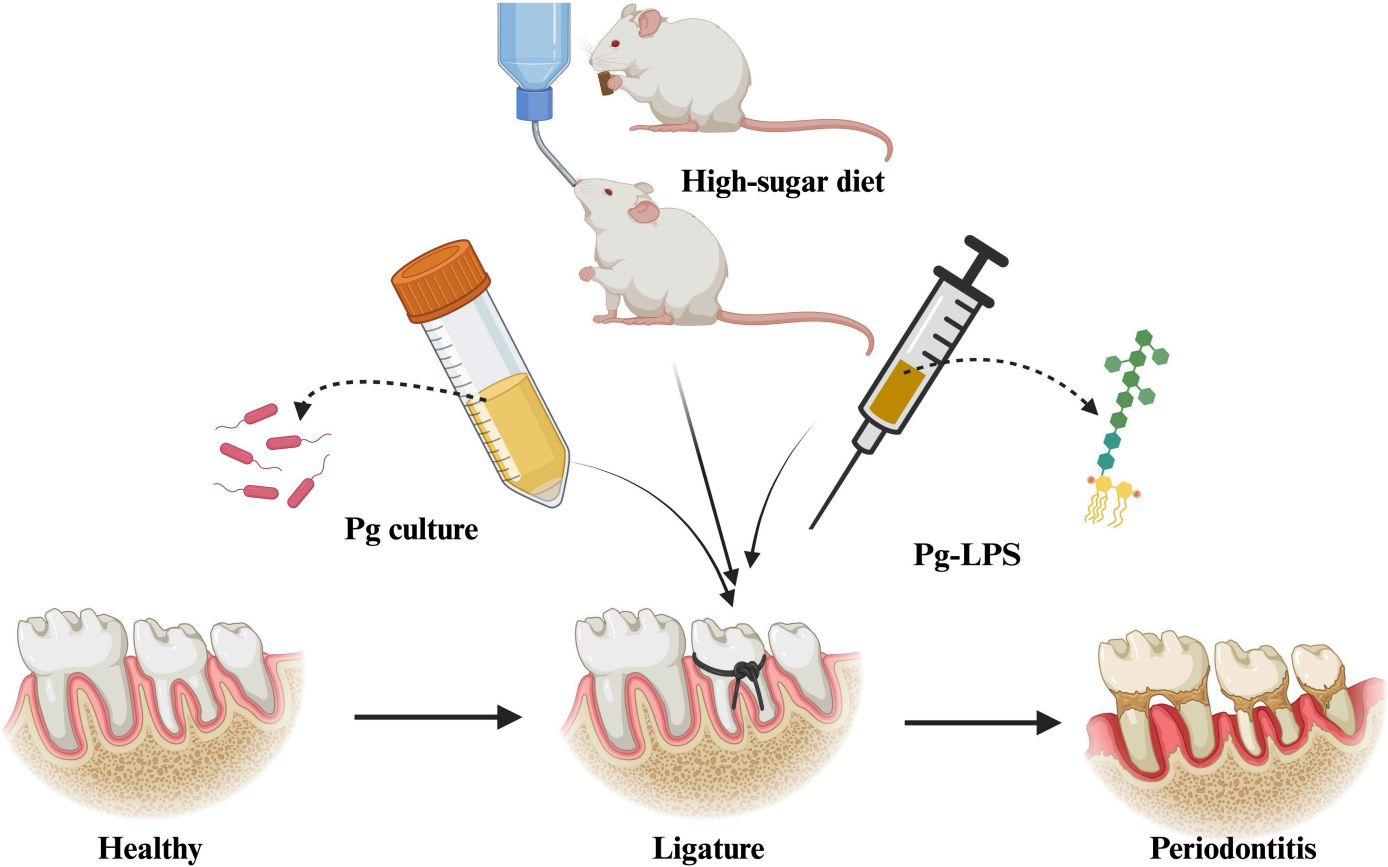
Schematic illustration of the multi-combination approach for constructing a periodontitis rat model.

The PD groups were fed a high-caries-inducing diet, Keyes 2000 (Nantong Trophy Feed Technology Co., Ltd.) and 10% sucrose water (Zhang et al., 2021). The NC group ate only standard chow and distilled water throughout the experiment, and the model group underwent ligation with 5-0 silk thread and given Pg-LPS and *P. gingivalis* culture once every 2 days. The BS-DS31 group received the bacterial suspension of DS31, the CFS-DS31 group was administered the fermentation supernatant of DS31, and the PC group was treated with compound chlorhexidine mouthwash. The schematic representation of the periodontitis model is presented in Figure 2.

### 2.7 Micro-computerized tomography (Micro-CT)

Maxillae were evaluated using a microcomputed tomography (micro-CT) system (Quantum GX2; PerkinElmer, Hopkinton, MA, USA). Before scanning, the maxillae were rinsed with double distilled water and transferred to sterile phosphate-buffered saline (PBS) then the micro-CT parameters were set as follows: voltage, 90 kV; current, 88 μA; field of view, 18 μm; acquisition time, 14 min; and camera mode, high resolution.

The following parameters were analyzed: (1) CEJ-ABC distance: the linear measurement from the cementoenamel junction (CEJ) to the alveolar bone crest (ABC) at both the distal and mesial aspects of the upper second molar (2) Bone mineral density: the concentration of minerals per unit volume of bone.

### 2.8 Histopathologic analysis

Samples were fixed in an Eppendorf tube using a 10% neutral buffered formalin solution. The jaw samples were subsequently decalcified for 24 h using a 10% formic acid solution. Following decalcification, the samples were dehydrated through a graded alcohol series (70%, 90%, 95%, and 99%) and cleared in xylene for 10 min before being embedded in paraffin. Sections of 5 μm thickness were cut in the mesiodistal direction using a microtome and stained with hematoxylin-eosin (H&E) for microscopic analysis (Lestari et al., 2023).

### 2.9 Detection of TNF-α, IL-1β, IL-6 and IL-8 in the periodontal tissues

The periodontal tissue samples were homogenized in RIPA lysis buffer (Shanghai Enzyme-linked Biotechnology Co., Ltd., Shanghai, China) supplemented with 1% (v/v) protease inhibitor cocktail (Shanghai Enzyme-linked Biotechnology Co., Ltd., Shanghai, China). The total protein concentration was determined using a BCA protein assay kit (Biosharp) according to the manufacturer’s protocol, with bovine serum albumin (BSA) serving as the standard and assumed to be 100% pure. The concentration of TNF-α, IL-1β, IL-6 and IL-8 were subsequently quantified using enzyme-linked immunosorbent assay (ELISA) kits (Shanghai Enzyme linked Biotechnology Co., Ltd., Shanghai, China) following the manufacturer-provided protocols. The results are reported in terms of BSA equivalents.

### 2.10 Statistical analysis

All experiments were performed in triplicate and are presented as the mean ± standard deviation (SD). Statistical analysis was performed using GraphPad Prism (version 9.5; GraphPad Inc., San Diego, CA, USA), and Dunnett’s test was employed to assess the significance of differences between the control and sample groups, with a threshold of *p* < 0.05.

## 3 Results

### 3.1 The effect of biofilm formation assay

**FIGURE 3:**
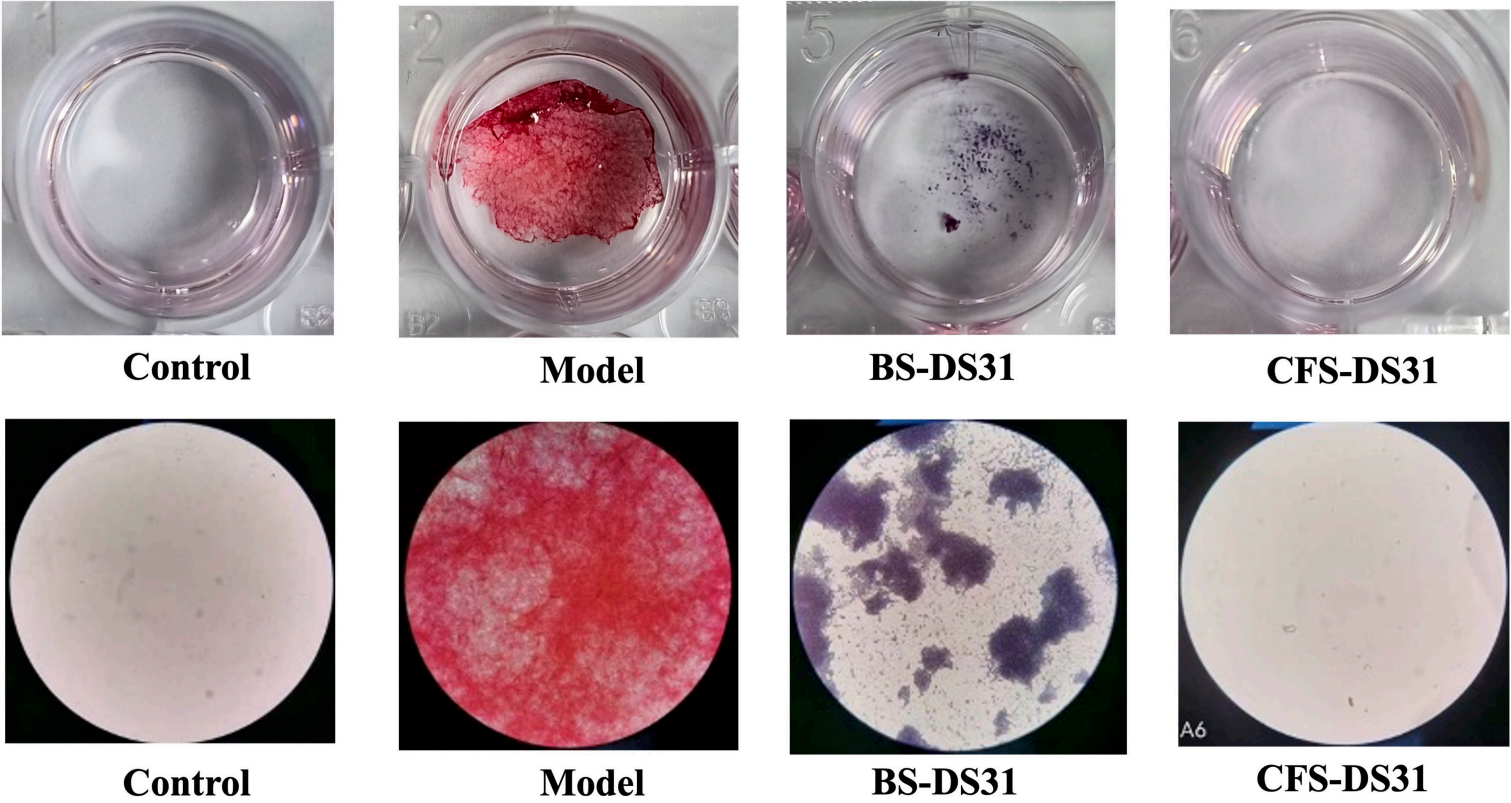
Microscopy images of *L. casei* DS31 inhibit *Porphyromonas gingivalis* biofilm formation stained with Gram stain after cultured.

The growth characteristics of the *P. gingivalis* biofilm were examined under a microscope. In the model group, a highly organized yet heterogeneous biofilm structure with numerous *P. gingivalis* organisms was observed. In the control group, only MRS Medium was added, and no biofilm was produced. The results demonstrated that both the BS-DS31 and the CFS-DS31 inhibited the formation of *P. gingivalis* biofilm. The purple coloration in the BS-DS31 group suggested that *L. casei* DS31 had colonized the biofilm and became the predominant bacterium. The effect observed in the CFS-DS31 was comparable to that of the blank control group. Both the BS-DS31 and the CFS-DS31 demonstrated significant inhibition of biofilm formation by *P. gingivalis* (Figure 3).

### 3.2 The effect of biofilm eradication assay

**FIGURE 4:**
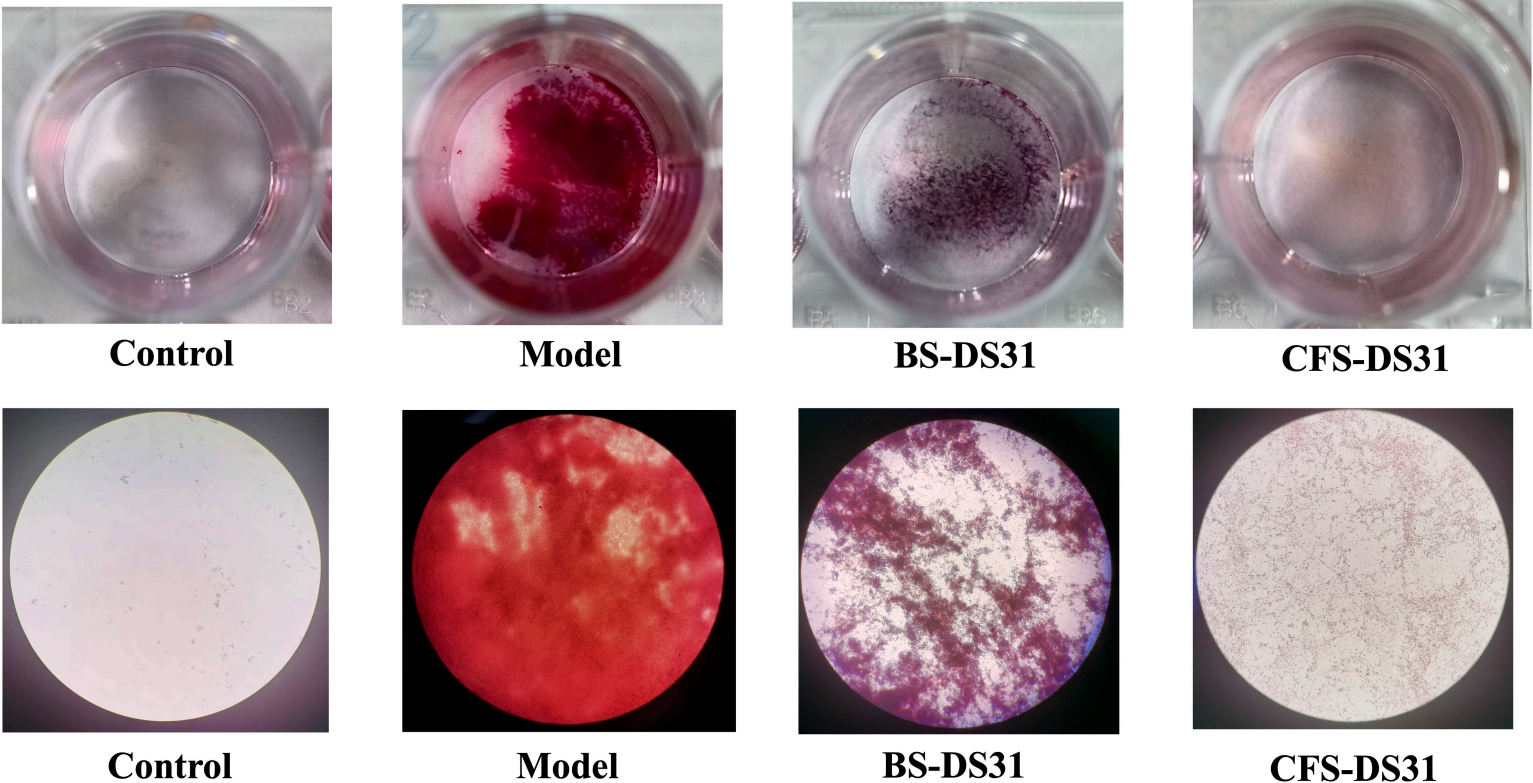
Microscopy images of mature *P. gingivalis* biofilm treated with *L. casei* DS31. Biofilm stained with Gram stain after cultured.

The mature *P. gingivalis* biofilm treated with BS-DS31 or CFS-DS31 was observed under the microscope. In the model group, after culturing *P. gingivalis* for 120 h, a highly organized yet heterogeneous biofilm structure containing numerous *P. gingivalis* organisms was observed. In the control group, only MRS medium was included. The results demonstrated that both the BS-DS31 and CFS-DS31 groups effectively cleared the mature *P. gingivalis* biofilm. Specifically, treatment with the BS-DS31 resulted in increased biofilm gaps and a purplish-red coloration, indicating the presence of a mixed biofilm of pathogenic bacteria and probiotics, with pathogenic bacteria being removed through co-aggregation. Following CFS-DS31 treatment, only lilac remnants were observed in the biofilm, suggesting the presence of antibacterial substances in the *L. casei* DS31 supernatant, which exhibited strong antibacterial activity (Figure 4).

### 3.3 The anti-inflammatory effects of *L. casei* DS31 on proinflammatory cytokine expression in *P. gingivalis* LPS-induced RAW 264.7 cells

**FIGURE 5:**
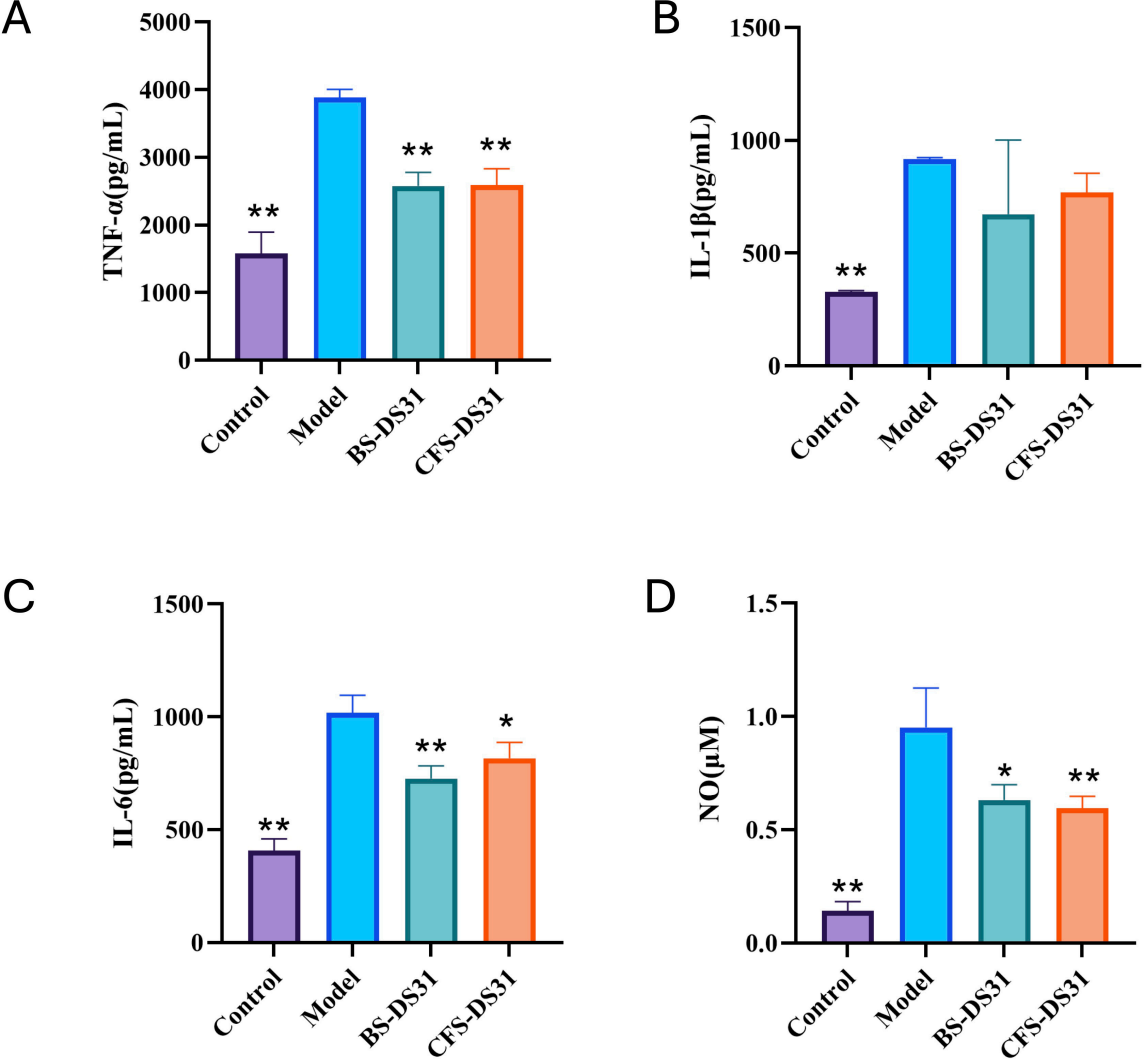
Effect of BS-DS31 or CFS-DS31 on proinflammatory cytokines in RAW264.7 cells. The concentrations of TNF-α (A), IL-1β (B) and IL-6 (C) were measured using ELISA kits. The levels of NO (D) were determined using assay kits. *p < 0.05, **p < 0.01 compared to the model group.

As anticipated, the exposure of RAW 264.7 cells to *P. gingivalis* LPS for 24 h clearly stimulated the cells and led to increased levels of proinflammatory cytokines (IL-6, IL-1β and TNF-α). However, treatment of RAW 264.7 cells by BS-DS31 or CFS-DS31 suppressed the levels of secreted and TNF-α, IL-1β and IL-6, after *P. gingivalis* LPS stimulation (Figures 5A–C), thereby mitigating the inflammatory response of RAW 264.7 cells. *P. gingivalis* LPS stimulation also significantly increased the secretion levels of the inflammatory mediator NO in RAW264.7. Notably, *L. casei* DS31 treatment effectively suppressed NO production following LPS exposure (Figure 5D).

### 3.4 Micro-CT analysis

**FIGURE 6:**
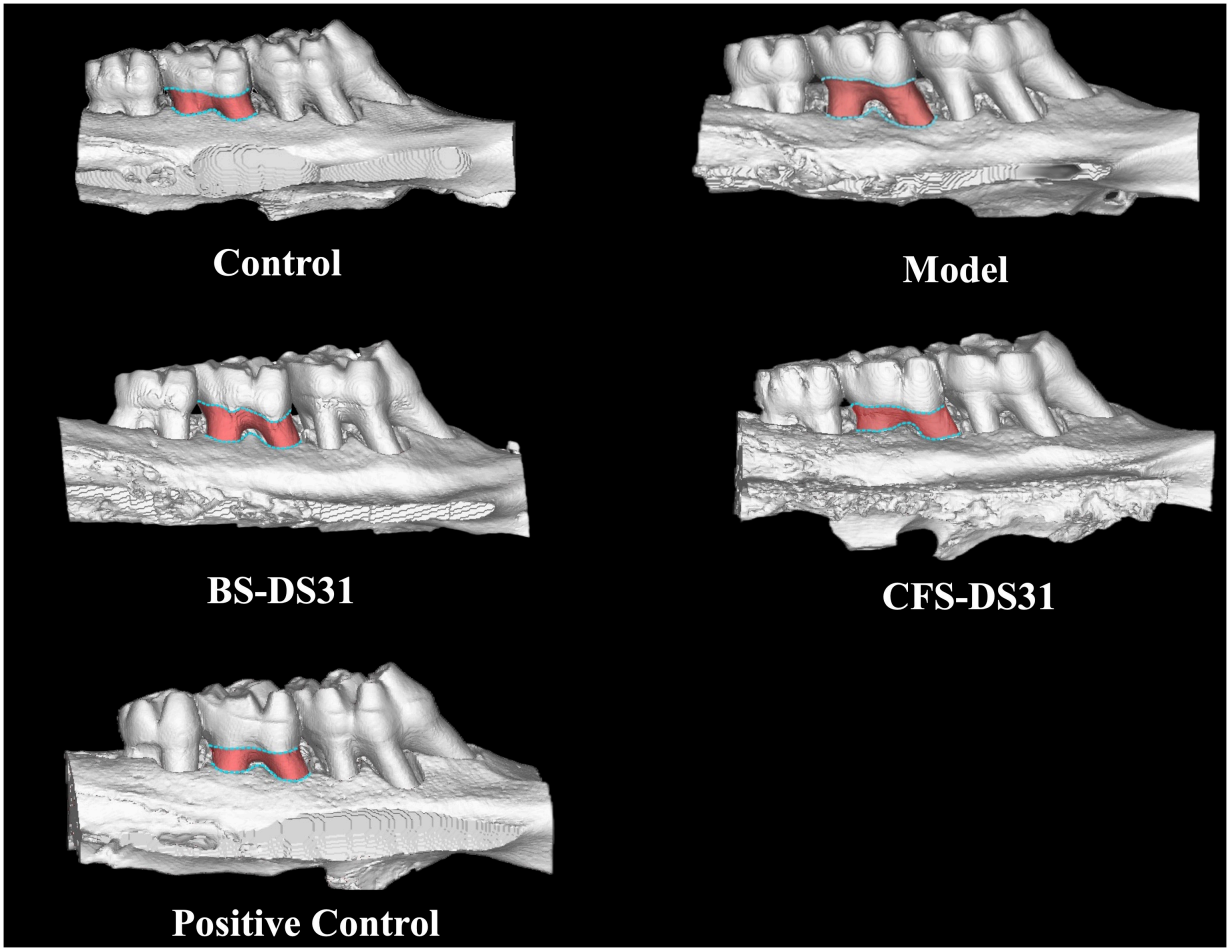
Micro-CT images of the maxillae.

**FIGURE 7:**
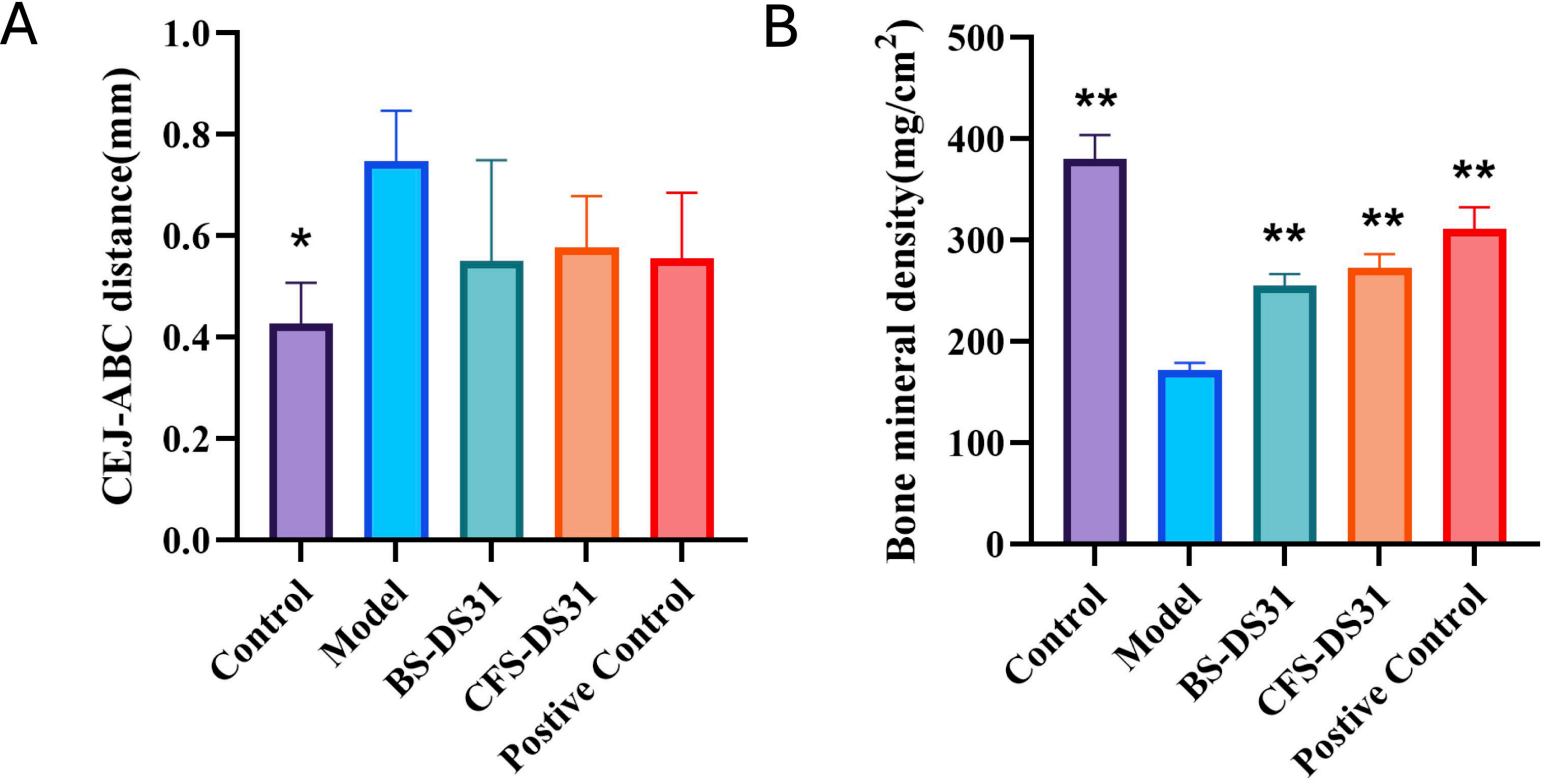
The cementoenamel junction-alveolar bone crest (CEJ-ABC) distance (A) and bone mineral density (BMD) (B) were used as indices of alveolar bone loss and were measured through micro-CT imaging. Data are represented as mean ± SD (*n* = 3). Significance was analyzed using Dunnett’s test. *p < 0.05, **p < 0.01 versus model group.

Periodontitis induces alveolar bone resorption in periodontal rats (Figure 6). In particular, the root furcation was obviously exposed, and the distance from the cementoenamel junction to the alveolar bone crest (CEJ-ABC; Figure 7A) increased significantly. In addition, the dental spaces in the model group were notably larger. Meanwhile, the bone density (Figure 7B) in the model group was significantly lower compared to that in the healthy group. The alveolar bone resorption in rats treated with BS-DS31 and CFS-DS31 was significantly reduced, as evidenced by a decreased CEJ-ABC distance compared to untreated periodontitis rats. Additionally, the alleviation of alveolar bone loss was observed through increased alveolar bone density, thereby enhancing the supportive function for teeth. Both BS-DS31 and CFS-DS31 can mitigate periodontitis in rats.

### 3.5 Histopathologic analysis

**FIGURE 8:**
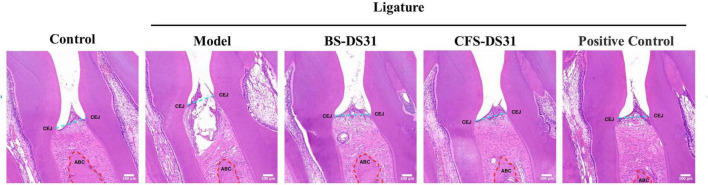
Representative H&E staining sections of gingival tissues. The scale bars represent 100 μm.

H&E staining revealed pronounced infiltration of highly proinflammatory immune cells, along with loss of connective tissue attachment and alveolar bone resorption between the distal root of the first molar and the mesial root of the second molar demonstrated by the distance of the cementoenamel junction (CEJ) to the alveolar bone crest (ABC), thereby clearly demonstrated that BS-DS31 and CFS-DS31 reduced periodontal tissue inflammation and apical movement of the junctional epithelium (Figure 8).

### 3.6 Immune response

**FIGURE 9:**
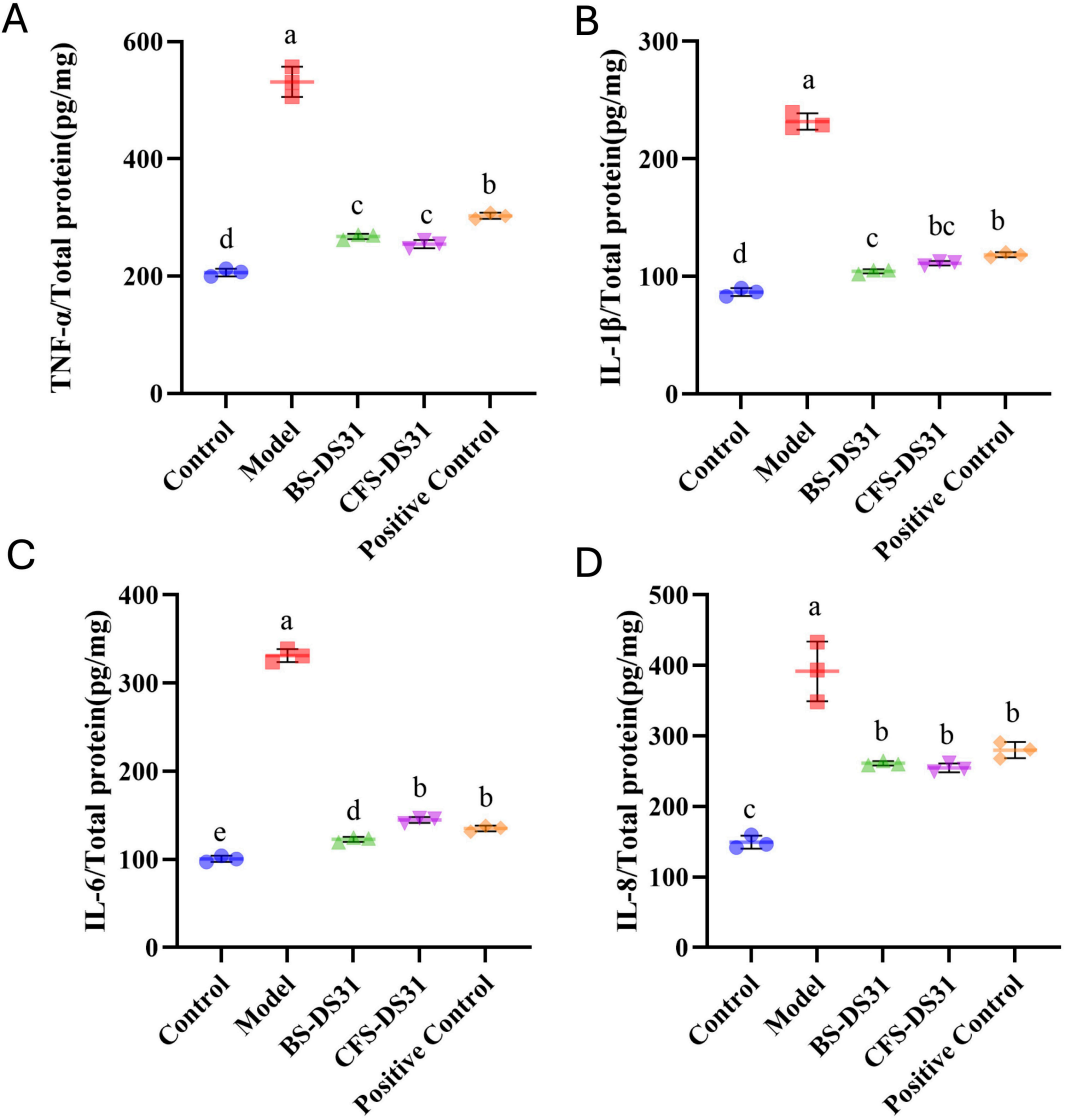
Effects of *L. casei* DS31 treatment on the expression of immune indicators TNF-α (A), IL-1β (B), IL-6 (C) and IL-8 (D). Groups with dissimilar letters differ, *p* < 0.05.

In addition to the effects on the composition of the oral plaque, damage to periodontal tissues is primarily mediated through changes in the inflammatory response. The levels of various inflammatory factors associated with periodontitis, including TNF-α, IL-1β, IL-6, and IL-8, were quantified using ELISA and were presented in Figure 8. After treatment with BS-DS31 and CFS-DS31, the levels of these four inflammatory factors were significantly reduced compared to those in the model group, indicating that both BS-DS31 and CFS-DS31 exert a beneficial effect on reducing gingival inflammation in periodontitis-induced rats (Figure 9).

## 4 Discussion

Periodontitis is a highly prevalent chronic inflammatory disease affecting individuals across all age groups. Bacterial biofilms, which adhere to surfaces such as human tooth enamel, consist of complex microbial communities enclosed within a self-produced extracellular polymeric substance (EPS) matrix. These structured communities enable free-living planktonic bacteria to transition into a multicellular mode of growth, contributing significantly to persistent and recurrent infections (Dsouza et al., 2024). Due to their dense architecture and protective matrix, biofilms pose a major challenge to conventional antimicrobial therapies, which often fail to penetrate and effectively eradicate the pathogens involved in periodontitis. The rising prevalence of antibiotic resistance, driven by the overuse of traditional antimicrobial agents, further underscores the need for more effective and innovative treatment strategies (Gerits et al., 2017). In response to these challenges, novel therapeutic approaches-including the use of probiotics-are currently being investigated to target and disrupt biofilm formation and enhance clinical outcomes.

To investigate and simulate the effects of probiotics on dental plaque, we established an *in vitro* periodontitis biofilm model to examine the preventive and therapeutic effects of BS-DS31 and CFS-DS31. The results demonstrated that both the BS-DS31 and CFS-DS31 exhibited significant inhibitory effects. After Gram staining, it was observed that in the BS-DS31 group, lactic acid bacteria formed a self-membrane and occupied the sites of the original *P. gingivalis* biofilm, while in the CFS-DS31 group, there was almost no presence of *P. gingivalis* biofilm. Notably, the BS-DS31 could auto-formed a biofilm, thereby displacing the *P. gingivalis* biofilm and becoming the dominant bacterium. In contrast, the CFS-DS31 almost completely inhibited the presence of pathogenic bacteria. Both *Lactobacillus* and *P. gingivalis* can form biofilms *in vitro*. To differentiate between these two bacteria, we utilized a Gram staining kit, which stained *P. gingivalis* red as gram-negative bacteria and *Lactobacillus* purple as gram-positive bacteria. Jiang et al. (2022) studied an antimicrobial peptide that exhibits potent inhibitory activity on both planktonic bacteria and biofilm of *P. gingivalis* and reduces the expression of its virulence factors.

In our study, *L. casei* DS31 was used in the form of a crude bacterial extract. However, according to the existing biofilm results, *L. casei* DS31 demonstrates significant inhibitory and scavenging effect on *P. gingivalis* biofilm. If future analyses can identify specific bacteriostatic compounds, such as proteins or bacterial polysaccharides, this would provide more concrete solutions for the prevention and treatment of periodontitis. It is widely recognized that the human oral environment is highly complex. Human oral cavity (mouth) hosts a complex microbiome consisting of bacteria, archaea, protozoa, fungi and viruses (Mosaddad et al., 2019). For example, bacterial species such as *P. gingivalis*, *Fusobacterium nucleatum*, *Actinomyces naeslundii*, *T. forsythia*, and *Streptococcus gordonii* play significant roles in this process (Kamińska et al., 2019). These bacteri are responsible for periodontitis. This disease is caused by plaques, which are a community of microorganisms in biofilm format. Future studies will aim to construct a more realistic model of periodontitis biofilm to evaluate the efficacy of *L. casei* DS31.

The immune system consists of both innate and adaptive immune responses. Innate immunity is responsible for recognizing and eliminating foreign pathogens during the early stages of infection, whereas adaptive immunity eliminates these pathogens through cytotoxic reactions and the secretion of antigen-specific antibodies. Macrophages, as a key component of the innate immune system, play a critical role in maintaining homeostasis by phagocytosing pathogens and releasing immunostimulatory mediators, including nitric oxide (NO), interleukin-1β (IL-1β), tumor necrosis factor-α (TNF-α), and interleukin-6 (IL-6; Park et al., 2023). The main pro-inflammatory factors are TNF-α, IL-6, and IL-1β, which are produced by macrophages and endothelial cells. IL-1β and TNF-α promote the aggregation and activation of inflammatory cells, stimulate the release of inflammatory mediators, induce fever, and amplify the inflammatory response. IL-6 stimulates macrophages to secrete monocyte chemoattractant protein-1 (MCP-1), thereby facilitating the migration of monocytes from blood vessels to sites of tissue inflammation (Luan et al., 2022). Therefore, reducing pro-inflammatory cytokines is crucial for alleviating inflammatory diseases. *In vitro*, lipopolysaccharide (LPS) from *P. gingivalis* was used to induce mouse macrophage RAW264.7 cells as a periodontitis model. BS-DS31 and CFS-DS31 were added to assess the secretion levels of inflammatory factors including TNF-α, IL-1β, and IL-6. The results demonstrated that the levels of TNF-α, IL-1β, and IL-6 were significantly reduced in the periodontitis cell models, indicating that DS31 exhibits potent anti-inflammatory effects. Wang Q. et al. (2020) demonstrated that DAA inhibited the secretion of NO, IL-1, IL-6, and TNF-α in LPS-induced RAW264.7 cells, indicating anti-inflammatory effects that are consistent with our findings. Yu et al. (2019) demonstrated that heat-inactivated JW15 exhibits anti-inflammatory properties through the inhibition of the NF-κB signaling pathway and modulation of the MAPK signaling pathway.

The animal experiment results demonstrated that *L. casei* DS31 significantly mitigated alveolar bone resorption and loss in rats, while reducing the secretion levels of inflammatory cytokines such as TNF-α, IL-1β, IL-6, and IL-8 in gingival tissues. The CEJ-ABC distance serves as an indicator of alveolar bone resorption. A greater CEJ-ABC distance signifies a larger exposed surface area of the alveolar bone, indicating more severe periodontitis. Following treatment with the DS31 bacterial solution and supernatant, the CEJ-ABC distance was reduced, with the BS-DS31 bacterial solution demonstrating slightly superior efficacy compared to the CFS-DS31. The bone loss in periodontitis-induced rats treated with *L. casei* DS31 was alleviated, as evidenced by the recovery of bone mineral density. This suggests that both the BS-DS31 and CFS-DS31 can enhance the tooth-supporting capacity in periodontitis-induced rats, thereby reducing the risk of tooth loss. This finding is consistent with the study by Lucateli et al. (2024), which offers novel insights into elucidating *L. casei* DS31’s role in osteoporosis for future research. In a periodontitis rat model, *Bifidobacterium longum* BL986 and *Lactobacillus rhamnosus* LRH09 were administered both individually and in combination. The combination treatment resulted in a significantly smaller infiltrated area of gingival cells and a reduced distance from the cementoenamel junction (CEJ) to the epithelial attachment (Chen et al., 2023). *L. casei* DS31 exhibits comparable effects on gingival tissues, and this study offers a novel perspective on utilizing probiotics synergistically for the prevention and treatment of periodontitis. Nakajima et al. (2021) reported a deep eutectic antibacterial agent (IDEA) ionic gel that simultaneously exhibited deep tissue penetration and antibacterial activity against *P. gingivalis*. This finding offers valuable insights for the subsequent development of probiotic drug delivery systems.

Due to technological and temporal constraints, our study requires further refinement and investigation. For example, the oral cavity features a complex and dynamic microenvironment, necessitating the development of a biofilm model that more closely mimics *in vivo* conditions, as well as the conduct of clinical trials to assess the preventive and therapeutic efficacy. Furthermore, the mechanism of action of BS-DS31 and CFS-BS31 remains unclear, warranting additional research into associated antibacterial compounds and signaling pathways.

## 5 Conclusion

The results demonstrated that *L. casei* DS31 can inhibit the growth of unformed *P. gingivalis* and eliminate preformed *P. gingivalis*, thereby reducing the secretion levels of inflammatory factors (TNF-α, IL-1β and IL-6) and mediators (NO) in inflammation-inducing cells. Through the establishment of a periodontitis animal model, it was found that DS31 can mitigate alveolar bone microstructural bone loss and resorption in periodontitis and reduce the inflammatory factors (TNF-α, IL-1β, IL-6 and IL-8) of gingival tissues. Our results strongly suggest that *L. casei* DS31 has the potential to act as a therapeutic agent or functional food for the prevention and treatment of periodontitis.

## Data Availability

The raw data supporting the conclusions of this article will be made available by the authors, without undue reservation.
